# Assessing Interpersonal Guilt Among Clinical Nurses in China: Cultural Adaptation and Psychometric Validation of the Interpersonal Guilt Rating Scale‐20

**DOI:** 10.1155/jonm/2792011

**Published:** 2026-09-22

**Authors:** Yu Shuang Chen, Yi Li, Qing Yang, Yang Yang, Fang Ma, Qiu Lan Hu, Lan Ding, Wei Wei, Yan Li, Yang Juan Bai

**Affiliations:** ^1^ The First Department of Cardiology, The First Affiliated Hospital of Kunming Medical University, Kunming, Yunnan, China, kmmc.cn; ^2^ Nursing Department, The First Affiliated Hospital of Kunming Medical University, Kunming, Yunnan, China, kmmc.cn; ^3^ Geriatric Intensive Care Unit, The First Affiliated Hospital of Kunming Medical University, Kunming, Yunnan, China, kmmc.cn; ^4^ Outpatient Department, The First Affiliated Hospital of Kunming Medical University, Kunming, Yunnan, China, kmmc.cn; ^5^ Department of Gastrointestinal Surgery, The First Affiliated Hospital of Kunming Medical University, Kunming, Yunnan, China, kmmc.cn; ^6^ Department of Obstetrics, The First Affiliated Hospital of Kunming Medical University, Kunming, Yunnan, China, kmmc.cn

**Keywords:** clinical nurse, interpersonal guilt, psychometric evaluation, reliability, scale, validity

## Abstract

**Background:**

Working in a chronically high emotional exhaustion environment, clinical nurses are prone to psychological burnout and associated professional psychological risks. Interpersonal guilt is recognized as an important potential mechanism affecting nurses’ mental health, which may impair their personal well‐being and nursing quality. However, there is still a lack of applicable assessment tools for measuring interpersonal guilt among Chinese clinical nurses.

**Objectives:**

The aim of this study is to translate the Interpersonal Guilt Rating Scale‐20 (IGRS‐20) into Chinese and evaluate its psychometric properties among clinical nurses in China.

**Research Design:**

A cross‐sectional research design.

**Participants and Research Context:**

A total of 451 Chinese clinical nurses were recruited using the convenience sampling method.

**Methods:**

The IGRS was translated into Chinese via the Brislin back‐translation method. Its reliability was assessed by internal consistency, split‐half, and retest reliability; validity was evaluated using content validity indicators, exploratory factor analysis (EFA), and confirmatory factor analysis (CFA).

**Results:**

The Chinese version of IGRS contained 18 items. Its Cronbach’s α coefficient was 0.833, with subscale values ranging from 0.780 to 0.831; split‐half reliability was 0.860 and retest reliability was 0.868. The scale’s I‐CVI ranged from 0.833 to 1.000, and S‐CVI was 0.981. The KMO value was 0.827, and Bartlett’s sphericity test yielded χ^2^ = 1524.165 (df = 153, *p* < 0.001). EFA adopting unweighted least squares extraction combined with Promax oblique rotation identified a robust four‐factor model explaining 52.095% of total common variance (factor loadings: 0.569–0.957). CFA confirmed good model fit (χ^2^/df = 1.432, CFI = 0.959, IFI = 0.960, GFI = 0.917), validating the model’s effectiveness for assessing nurses’ interpersonal guilt.

**Conclusion:**

The Chinese IGRS has good reliability and validity among Chinese clinical nurses. It can be used to measure nurses’ interpersonal guilt level, facilitate the understanding of their psychological state, lay a foundation for targeted mental health interventions, and ultimately improve nursing quality.

**Implications for Nursing Management:**

The Chinese version of the IGRS provides a reliable assessment tool for nursing management to identify clinical nurses with high interpersonal guilt, enabling the design of culturally tailored psychological support programs that enhance nurses’ emotional resilience and reduce professional psychological risks, thereby stabilizing the nursing workforce.

## 1. Introduction

Nursing is a profession that demands sustained emotional investment, with its core responsibility centered on the lives and well‐being of others [1, 2]. While the intense sense of duty and empathy required endows the work with meaning, it simultaneously exposes nurses to unique psychological stressors [3]. In the contemporary healthcare environment, characterized by increasingly complex patient conditions and ever‐growing care demands, these occupational and emotional pressures are continually amplified [3, 4]. Data from the World Health Organization indicate that over 40% of nurses report experiencing high‐intensity workloads and psychological burnout [5], which in turn precipitates professional psychological risks such as compassion fatigue and moral distress [6]. These risks not only directly compromise the quality of care but also severely erode nurses’ personal well‐being and professional quality of life [7–9]. Research suggests that guilt, as a prevalent negative emotion, is a significant factor contributing to this array of psychological challenges among nurses [1, 10, 11]. It not only directly triggers emotional distress but also, by intensifying self‐blame and perpetuating emotional exhaustion, serves as a potential mechanism for the development and exacerbation of psychological issues among nurses [12].

Guilt is commonly conceptualized as a self‐conscious moral emotion arising from the perception of having violated personal or social standards, often accompanied by feelings of regret, remorse, and a desire to make amends [13, 14]. Notably, the generation of guilt is often closely associated with interpersonal interactions. Especially in professions like nursing that are highly dependent on relational connections, guilt mostly manifests in the form of “interpersonal guilt” [15]. Interpersonal guilt is a prosocial and altruistic emotion grounded in cognitions associated with loyalty, empathy, and care for others [16]. The development of healthy interpersonal guilt may originate from prior erroneous behavior and foreshadow positive options in the future [17]. However, when cognitive distortions related to interpersonal guilt become excessive, this emotion can evolve into pathogenic interpersonal guilt [17]. Pathogenic interpersonal guilt encompasses the following distinct subtypes: survival guilt, separation/disloyalty guilt, omnipotent responsibility guilt, self‐hate guilt, and burdening guilt [18].

These pathogenic subtypes are particularly relevant to clinical nurses, whose work is characterized by high empathy, heavy responsibility, and frequent interpersonal conflicts, rendering them especially susceptible to such pathological guilt [1]. Failure to manage this guilt can result in serious psychological problems and even mental illness [11]. Bryan and O’Connor’s studies showed that guilt was closely related to the appearance of depression [19, 20]. Qaderi Bagajan [21]found a close association between guilt and suicidal tendencies in a study on individual suicidal behavior. Furthermore, some studies have confirmed the positive associations between interpersonal guilt and low self‐esteem [22], anxiety symptoms [23], post‐traumatic stress symptoms [24], and low levels of well‐being [25]. Guilt can also, to varying extents, reduce the quality of nurses’ clinical work. Specifically, guilt might make it arduous for nurses to concentrate during work and diminish their decision‐making capabilities, thereby impinging upon the quality of patient care [12, 26]. In addition, guilt may also lead nurses to overblame themselves and affect their professional judgment and clinical practice [12, 26]. However, despite the established global evidence on its detrimental effects, interpersonal guilt remains critically understudied among nurses in China, largely due to the absence of a validated assessment tool.

The Interpersonal Guilt Rating Scale (IGRS‐20), developed by Leonardi et al. [18], offers a promising multidimensional measure for assessing this construct, yet its applicability in Chinese healthcare settings remains unclear. Although the IGRS has been increasingly utilized in recent years, most existing studies have been conducted in Western populations. Empirical evidence has demonstrated that interpersonal guilt is closely associated with a range of transdiagnostic psychological constructs. Leonardi et al. [27] found that survivor guilt, separation/disloyalty guilt, and omnipotent responsibility guilt were significantly associated with attachment anxiety and avoidance, altruism, and personality pathology, with self‐hate guilt showing the strongest unique association with personality dysfunction. In the context of psychotherapy, Doorn et al. [28] revealed that childhood adverse experiences significantly moderated the effect of interpersonal guilt on the working alliance, suggesting that interpersonal guilt may influence patients’ capacity for mentalization and trust in the therapeutic relationship. Fiorenza et al. [29] further reported that interpersonal guilt activation was heightened during conflictual interactions compared to nonconflictual ones, indicating that interpersonal guilt is closely tied to relational frustration and tolerance for interpersonal tension. Neurocognitive studies have also identified an early frontal negativity associated with guilt processing emerging at approximately 350–500 ms following feedback, providing neural evidence for the temporal dynamics of guilt‐related cognitive processing. Within the Chinese context, Wang et al. [30] found that survivor guilt predicted depressive symptoms among college students during the COVID‐19 quarantine, with intrusive rumination serving as a mediating mechanism and deliberate rumination acting as a protective factor. Zhang [31] further examined the social value of interpersonal guilt in maintaining social norms and promoting prosocial behavior. However, none of these Chinese studies employed the IGRS, and research on interpersonal guilt among Chinese clinical nurses remains virtually absent. Therefore, the present study aimed to translate the IGRS into Chinese and evaluate its psychometric properties among Chinese clinical nurses.

Furthermore, it is noteworthy that interpersonal guilt, as an emotion deeply rooted in social relationships and cultural cognition, varies significantly across cultural contexts [32]. Systematic differences between Eastern and Western cultures in self‐identity, attribution of responsibility, and emotional norms suggest that directly applying instruments developed in Western settings may introduce cultural bias [33]. Therefore, conducting systematic cross‐cultural adaptation and validation is essential to ensure the measurement equivalence and clinical applicability of such tools in the Chinese context. The IGRS‐20, developed by Leonardi et al. [18] in 2023, is a multidimensional tool designed to assess interpersonal guilt across specific psychological dimensions. Therefore, this study aims to translate and culturally adapt the IGRS‐20 into Chinese, as well as to examine the psychometric properties of this Chinese version among clinical nurses in China.

## 2. Methods

### 2.1. Study Design and Participants

A cross‐sectional survey was utilized to assess the Chinese version of the IGRS. From August 2024 to October 2024, 451 clinical nurses from tertiary hospitals in Yunnan Province were selected by convenient sampling. The inclusion criteria for participants were as follows: (1) full‐time registered nurses; (2) nurses who had at least one year of clinical practice experience; and (3) nurses who voluntarily engaged in the study and given signed informed consent. Exclusion criteria are as follows: (1) Being on leave (sick leave, maternity leave); (2) nonhospital nurses who come for advanced studies; (3) nursing intern (nursing intern is a nursing graduate who engages in nine months of clinical instruction at hospitals prior to taking the national nurse licensure examination and does not possess independent working privileges during this time); and (4) nurses with mental illness or severe physical illness.

In accordance with Kendall’s method for calculating sample size, the required sample size ought to be 5 to 10 times the quantity of questionnaire items. Therefore, considering that the IGRS consists of 20 items, our study required a sample size ranging from 100 to 200 participants. However, assuming a 20% attrition rate, the required sample size would be 120 to 240 individuals. This study recruited a total of 451 clinical nurses. During the survey, participants were provided with standardized instructions to explain the content and purposes of the study in detail. All participants were informed that their participation was of a voluntary nature, their responses would be kept confidential, and they had the right to withdraw at any time.

### 2.2. Instruments

#### 2.2.1. General Demographic Characteristics Questionnaire

Based on the research objective, the researchers formulated a questionnaire to gather general demographic details such as gender, age, departments, years of work experience, the frequency of night shifts per month, the quantity of patients rescued per month, educational attainment, marital status, and the number of children.

#### 2.2.2. IGRS

The IGRS was developed by Leonardi et al. [18] in 2023, consisting of 20 items covering four dimensions: self‐hate (3 items), survivor guilt (5 items), omnipotent guilt (7 items), and burdening guilt (5 items). The scale was evaluated on a 5‐point Likert scale, with 1 indicating (Very Uncharacteristic) and 5 representing (Very Characteristic). Cronbach’s alpha coefficients were adequate and good: 0.81 for self‐hate, 0.82 for survivor guilt, 0.77 for omnipotent guilt, and 0.73 for burdening guilt.

### 2.3. Procedures

#### 2.3.1. Scale Translation Procedure

With the explicit consent and authorization of the original scale development team members [18], we translated and adjusted the scale across cultures. IGRS was converted into Chinese by means of the classical “backwards and forward” approach in accordance with a modified Brislin translation model [34]. There were three steps in the translation process.

• Step 1: translation
 Two nursing graduate students with a good command of English carried out the translation of the IGRS scale and made an independent comparison of the two translated versions. A first draft of the Chinese version was generated following negotiation and discussion.
• Step 2: back‐translation
 Two experts who were highly proficient in English and had foreign learning experience but were unfamiliar with the original scale carried out the back‐translation. Both experts conducted the back‐translation individually. Subsequently, the resulting versions were compared and discussed to achieve a consensus. The final translated scale was proven to have consistency with the original version.
• Step 3: culture adaptation
 A group of experts was set up, which comprised six researchers, among them one psychologist and five nursing experts. The expert group made a comparison between the translation and back‐translation versions and the original scale. They took into account cultural adaptation and conceptual equivalence of idioms so as to make the language expression more conform to the language norms of the mainland. Then, using a convenient sampling method, 30 nurses were selected from a tertiary hospital in Kunming for a preliminary experiment. Upon completion of the scale, all participants affirmed that the topic was precise, the structure was robust, the logic was coherent, and the meaning was clear, while also providing suggestions for cultural refinement of the scale.

Finally, the Chinese version of the IGRS was revised and enhanced in light of experts’ opinions and preliminary experiment results. The details were as follows: Item 11, “I feel I should visit my parents as often as they would like,” and Item 13, “I feel I should put my parent’s wishes ahead of my own,” were deleted. In Chinese cultural traditions, filial piety is an extremely important concept. For every individual, visiting and loving parents are the basic requirements that we should follow. Therefore, the team decided to delete these two items. Finally, the Chinese version of the IGRS contained 18 items.

### 2.4. Data Collection

Participants in this study were selected by a convenient sampling method from a tertiary hospital in Kunming, Yunnan Province, China. The inclusion and exclusion criteria for formal data collection were consistent with the preliminary experiment. Upon obtaining the consent of the nursing department manager and the head nurses of relevant departments, data collection was carried out simultaneously via electronic questionnaires and paper questionnaires. Prior to disseminating the questionnaires, trained members of the research team elucidated the study’s goal and importance to the participants. The participants were assured that the study data would be utilized just for academic purposes and would not be divulged or employed for any other reasons without their consent. Uniform instructions were provided for the questionnaires, and completion on the same smart device was restricted to one time. The survey involved 471 clinical nurses. Among them, 451 valid questionnaires were gathered, and the effective recovery rate reached 95.8%. To evaluate the scale’s test–retest reliability, 30 nurses were chosen to participate in a second survey two weeks later.

### 2.5. Statistical Analyses

Statistical data analysis in this study was conducted using SPSS (V26.0) and AMOS (V26.0). Demographic characteristics were evaluated through descriptive statistics, with mean and standard deviation for continuous data and frequency and percentage for categorical variables. To compare IGRS dimensional scores across sociodemographic groups, independent‐sample *t* tests were used for dichotomous variables (gender and marital status), one‐way ANOVA for age, working years, and departments, and Kruskal–Wallis H tests for other categorical variables (professional title, educational level, night shifts, patient resuscitations, and number of children), with post hoc pairwise comparisons performed using the Bonferroni correction.

### 2.6. Items Analysis

The item analysis aimed to ascertain the scale’s differentiation and relevance. The top 27% of the scale’s total score constituted the high group, while the bottom 27% formed the low group. The difference and significance between the two groups on each item were calculated to judge the adequacy of the scale. A critical ratio (CR) over 3.00 signifies effective discriminating among entries [35]. The retention of each item in the Chinese version of the IGRS was evaluated by analyzing the item‐total correlation and the Cronbach’s alpha coefficient of the omitted item. The criterion for judging the item‐total correlation was set at 0.4 [36].

### 2.7. Validity Analysis

Validity analysis pertains to the degree to which a measurement instrument is capable of precisely gauging that which requires measurement [37]. This study focused on the examination of both content and structural validity. Using the expert consultation method, six experts were invited to evaluate the relevance of each item in the table to its respective dimensions. A 4‐point Likert scale was employed, where 1 denotes irrelevant and 4 denotes signified highly relevant. Content quality analysis makes use of the item‐level content validity index (I‐CVI) and the scale‐level content validity index (S‐CVI). When the I‐CVI exceeds 0.800 and the S‐CVI is greater than 0.900, the quantitative content validity is considered to be good [38].

The factor results of the IGRS were evaluated by means of exploratory factor analysis (EFA) and confirmatory factor analysis (CFA). The sample of 541 cases was randomly subdivided into two groups: one (*n* = 224) for EFA and the other (*n* = 227) for CFA. Considering the non‐normal distribution of sample data and the theoretical correlation between different interpersonal guilt dimensions, EFA adopted unweighted least squares (ULS) for factor extraction with Kaiser‐normalized Promax oblique rotation. AMOS software was used to implement CFA and test the goodness‐of‐fit indices of the theoretical model.

### 2.8. Reliability Analysis

To assess the reliability of the translated scale, Cronbach’sαcoefficients, split‐half reliability, and test–retest reliability were analyzed. Cronbach’s α coefficients for the overall scale and each dimension were computed, and a value of 0.70 was taken as the acceptable standard for the reliability coefficient [39]. Split‐half reliability was evaluated by partitioning the scale items into two equal halves in sequential order and computing the correlation between the scores of the two halves. Two weeks later, 30 clinical nurses were retested with the translated scale. The correlation between the retest results and the first measurement was analyzed to assess stability and consistency during data collection.

### 2.9. Ethical Considerations

This study protocol was approved by the Ethics Committee of the First Affiliated Hospital of Kunming Medical University on July 9, 2024 (Approval No. 2024L167). The methods utilized in this study are in accordance with the principles set forth in the *Declaration of Helsinki*. All participants completed an informed consent form before filling out the questionnaire. In addition, participants were assured that their participation in this study was voluntary. They were also informed that the data derived from their responses would be used solely for academic research purposes.

## 3. Results

### 3.1. Descriptive Statistics

This study collected 471 questionnaires. After eliminating 20 invalid questionnaires, 451 valid questionnaires were ultimately obtained, including 416 (92.2%) female nurses and 35 (7.8%) male nurses, with 45% of them in the age range of 26–35 years. Detailed characteristics of demographic data are presented in Table 1.

**TABLE 1:** Frequency distribution of demographic characteristics (*n* = 451).

Factors	Group	*n*	%
Gender	Male	35	7.80
	Female	416	92.20

Age	≤ 25	98	21.70
	26–35	203	45
	36–45	120	26.60
	≥ 46	30	6.70

Departments	Internal medicine	219	48.60
	Surgery	131	29
	Emergency department	15	3.30
	Obstetrics, gynecology, and pediatrics	14	3.10
	Intensive care unit	27	6
	Other departments	45	10

Professional title	Primary nurse	84	18.60
	Nurse practitioner	154	34.10
	Nurse‐in‐charge or above	213	47.20

Working years	1–5	162	35.90
	6–10	97	21.50
	11–15	76	16.90
	16–20	58	12.90
	> 20	58	12.90

The number of night shifts per month	0	107	23.70
	1–4	102	22.60
	5–8	172	38.10
	> 8	70	15.50

The number of patients rescued per month	0	125	27.70
	1–3	250	55.40
	4–6	50	11.10
	6–10	15	3.30
	> 10	11	2.40

Educational level	Junior college degree or below	21	4.60
	Undergraduate education	408	90.50
	Postgraduate education or above	22	4.90

Marital status	Unmarried	179	39.70
	Married	272	60.30

Number of children	0	185	41
	1	144	31.90
	≥ 2	122	27

### 3.2. Item Analysis

The CR values for the 18 scale items varied from 8.307 to 16.501, with statistically significant differences between the high‐score and low‐score groups (*p* < 0.001). Each item exhibited a positive correlation with the total score of the scale, with the correlation coefficients ranging from 0.486 to 0.632 (*p* < 0.001), and all of them were greater than 0.400. After the deletion of each item, the Cronbach’s α coefficients of the Chinese version of the IGRS ranged from 0.817 to 0.829, remaining below the original Cronbach’s α coefficient of 0.833 (Table 2).

**TABLE 2:** The result of item analysis of IGRS (*n* = 451).

Item	Low‐score group (*N* = 130)	High‐score group (*N* = 123)	*t*	*p*	Item‐total correlation	Cronbach’s alpha if item deleted
	Mean ± SD	Mean ± SD				
1	1.93 ± 1.06	3.51 ± 1.28	10.84	< 0.001	0.489	0.828
2	1.71 ± 0.72	3.78 ± 1.25	16.408	< 0.001	0.600	0.820
3	1.86 ± 0.72	3.69 ± 1.27	14.421	< 0.001	0.585	0.821
4	1.71 ± 0.79	3.88 ± 1.28	16.501	< 0.001	0.632	0.817
5	2.06 ± 0.74	3.48 ± 1.10	12.206	< 0.001	0.486	0.826
6	1.91 ± 0.82	3.32 ± 0.91	13.202	< 0.001	0.549	0.822
7	2.17 ± 0.88	3.41 ± 0.90	11.291	< 0.001	0.550	0.824
8	2.12 ± 0.81	3.23 ± 1.02	9.72	< 0.001	0.551	0.829
9	2.08 ± 0.89	3.18 ± 1.01	9.443	< 0.001	0.552	0.826
10	2.06 ± 0.83	3.30 ± 1.08	10.445	< 0.001	0.553	0.825
12	1.96 ± 0.83	3.41 ± 0.99	12.753	< 0.001	0.554	0.821
14	2.05 ± 0.95	3.22 ± 0.99	9.699	< 0.001	0.555	0.824
15	2.20 ± 0.82	3.12 ± 0.96	8.307	< 0.001	0.556	0.828
16	2.12 ± 0.89	3.26 ± 0.83	10.707	< 0.001	0.557	0.825
17	2.11 ± 0.80	3.35 ± 1.01	11.017	< 0.001	0.558	0.827
18	1.98 ± 0.87	3.26 ± 0.98	11.205	< 0.001	0.559	0.825
19	2.09 ± 0.78	3.43 ± 0.95	12.509	< 0.001	0.560	0.824
20	2.05 ± 0.91	3.22 ± 0.91	10.409	< 0.001	0.561	0.827

### 3.3. Validity Analysis

#### 3.3.1. Content Validity Analysis

Six experts were engaged to assess the significance of each item within the scale. The evaluation findings demonstrated that the I‐CVI score fluctuated from 0.833 to 1.000, and the S‐CVI value was 0.981.

#### 3.3.2. Construct Validity Analysis

##### 3.3.2.1. EFA

**FIGURE 1:**
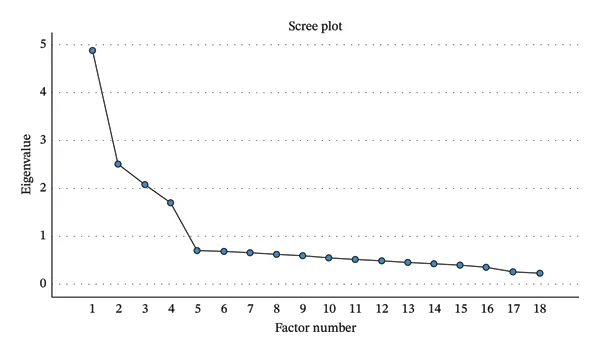
Screen plot of exploratory factor analysis for the Chinese version of the IGRS.

The KMO value was 0.827, exceeding the recommended threshold of 0.800, and Bartlett’s sphericity test was significant (*χ* ^2^ = 1524.165, df = 153, *p* < 0.001), indicating that the data were suitable for EFA. Using the ULS extraction method with Promax oblique rotation, four factors with eigenvalues greater than 1 were retained, cumulatively explaining 52.095% of the total variance based on the extraction sums of squared loadings. Since all items exhibited factor loadings exceeding 0.50 on their respective primary factors, no items were deleted. The factor loadings derived from the pattern matrix are presented in Table 3. The scree plot further confirmed the four‐factor structure (Figure 1).

**TABLE 3:** Exploratory factor analysis (*n* = 224).

Item	F1	F2	F3	F4
Q3	**0.888**	0.091	−0.089	−0.016
Q5	**0.716**	0.023	−0.022	−0.05
Q9	**0.681**	−0.068	−0.003	0.046
Q14	**0.679**	−0.114	0.123	0.02
Q20	**0.603**	0.023	0.033	−0.012
Q2	−0.025	**0.814**	0.069	−0.065
Q19	−0.005	**0.657**	0.011	0.072
Q16	−0.082	**0.645**	0.105	−0.037
Q6	0.119	**0.683**	−0.182	0.053
Q10	−0.046	**0.702**	0.013	0.02
Q17	0.154	0.084	**0.604**	−0.007
Q18	−0.059	0.043	**0.596**	0.098
Q7	0.168	0.021	**0.569**	−0.009
Q4	−0.094	0.019	**0.728**	−0.073
Q12	−0.021	−0.108	**0.73**	0.046
Q1	0.033	−0.023	−0.044	**0.957**
Q8	−0.068	0.021	0.028	**0.763**
Q15	0.026	0.091	0.063	**0.624**

*Note:* F1 = burdening guilt; F2 = survival guilt; F3 = omnipotent guilt; F4 = self‐hate guilt. Bold values represent the primary factor loading of each item. Factor loadings > 0.50 indicate that the item belongs to the corresponding factor dimension.

##### 3.3.2.2. CFA

**FIGURE 2:**
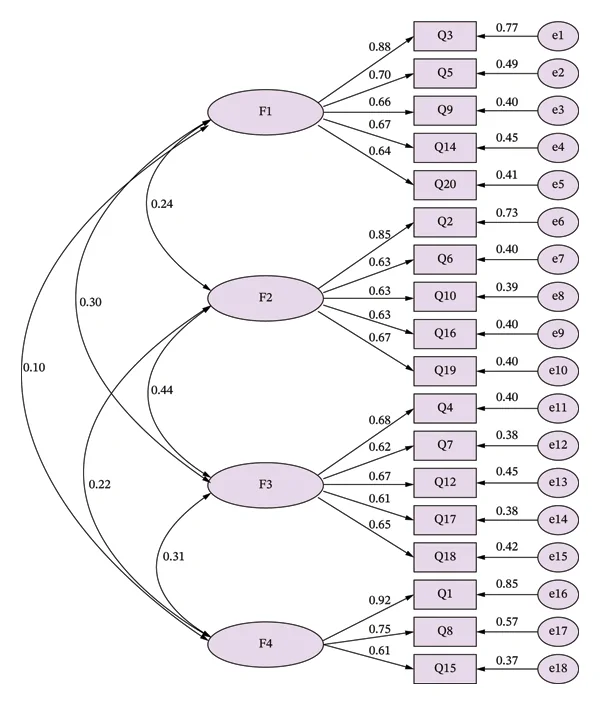
Diagram of the standardized four‐factor structural validity model of the Chinese version of the IGRS.

The results of CFA offered support for the four‐factor structure of IGRS‐18. The model fitting indices, including *X* ^2^/df = 1.432, RMSEA = 0.044, and GFI = 0.917, fell within the acceptable range. This is shown in Table 4 and Figure 2. All the fitting indices indicated that the model employed in this study fitted well with the data used.

**TABLE 4:** Evaluation fitness of the IGRS model.

Model	*X* ^2^/df	RMSEA	GFI	NFI	TLI	CFI	IFI	PNFI
Model modification	1.432	0.044	0.917	0.879	0.952	0.959	0.960	0.741
Standard value	< 3.000	< 0.080	> 0.900	> 0.900	> 0.900	> 0.900	> 0.900	> 0.500

### 3.4. Reliability Analysis

Cronbach’s α coefficient of the translated scale was 0.833, and the values of the four dimensions ranged from 0.780 to 0.831. The split‐half reliability value was 0.860. Two weeks later, 30 clinical nurses were selected for retest, and the retest reliability value was 0.868 (Table 5).

**TABLE 5:** Reliability analysis for the Chinese version of the IGRS.

The scale and its dimension	Cronbach’s alpha	Split‐half reliability	Test‐retest reliability
Interpersonal Guilt Rating Scale	0.833	0.860	0.868
Survival guilt	0.817		
Omnipotent guilt	0.780		
Self‐hate guilt	0.810		
Burdening guilt	0.831		

### 3.5. Differences in IGRS Scores Among Nurses With Different Demographic Characteristics

Independent‐samples t test was adopted to compare IGRS dimensional scores of gender and marital status. For multicategory demographic indicators with three or more subgroups including age, departments, professional title, working years, the number of night shifts per month, the number of patients rescued per month, educational level, and the number of children, one‐way ANOVA was adopted when the dimensional scores met normal distribution and homogeneity of variance; otherwise, the Kruskal–Wallis H test was used to analyze intergroup differences of the four IGRS factor scores.

As presented in Table 6, no significant differences in all IGRS dimensional scores were detected in terms of gender, age, departments, professional title, working years, the number of night shifts per month, the number of patients rescued per month, marital status, and the number of children (all *p* > 0.05). A statistical difference was merely found in the self‐hate guilt dimension across different educational level groups (H = 6.171, *p* = 0.046). Further pairwise comparison indicated that nurses with junior college degree or below had significantly lower self‐hate guilt scores than nurses with postgraduate education or above, and no remarkable differences existed among other educational subgroups.

**TABLE 6:** Comparison of four‐factor scores among different demographic groups (*n* = 451).

Variable	Group	Burdening guilt (M ± SD)	Survival guilt (M ± SD)	Omnipotent guilt (M ± SD)	Self‐hate guilt (M ± SD)
Gender	Male	2.84 ± 1.02	2.76 ± 0.83	2.53 ± 0.75	2.64 ± 0.85
	Female	2.69 ± 0.87	2.65 ± 0.84	2.74 ± 0.83	2.76 ± 1.01
	*t*	0.966	0.758	−1.462	−0.779
	*p*	0.335	0.449	0.144	0.440

Age	≤ 25	2.74 ± 0.93	2.57 ± 0.80	2.67 ± 0.83	2.72 ± 0.95
	26–35	2.63 ± 0.89	2.71 ± 0.87	2.76 ± 0.81	2.70 ± 1.01
	36–45	2.78 ± 0.83	2.66 ± 0.83	2.70 ± 0.85	2.83 ± 1.05
	≥ 46	2.77 ± 0.94	2.61 ± 0.76	2.81 ± 0.80	2.87 ± 0.86
	*p*	0.802	0.509	0.503	0.658

Departments	Internal medicine	2.63 ± 0.88	2.65 ± 0.82	2.78 ± 0.87	2.77 ± 1.03
	Surgery	2.76 ± 0.86	2.60 ± 0.87	2.72 ± 0.74	2.68 ± 1.00
	Emergency department	2.73 ± 1.07	2.87 ± 0.70	2.65 ± 0.82	3.13 ± 0.93
	Obstetrics, gynecology, and pediatrics	2.63 ± 0.99	2.30 ± 0.78	2.47 ± 0.72	2.93 ± 0.96
	Intensive care unit	2.74 ± 0.87	2.96 ± 0.87	2.49 ± 0.77	2.67 ± 0.98
	Other departments	2.87 ± 0.89	2.70 ± 0.82	2.76 ± 0.89	2.67 ± 0.90
	*F*	0.712	1.537	0.906	0.767
	*p*	0.614	0.177	0.477	0.574

Professional title	Primary nurse	2.69 ± 0.93	2.59 ± 0.80	2.69 ± 0.79	2.69 ± 0.90
	Nurse practitioner	2.65 ± 0.87	2.67 ± 0.82	2.71 ± 0.82	2.76 ± 1.07
	Nurse‐in‐charge or above	2.74 ± 0.88	2.67 ± 0.86	2.75 ± 0.85	2.76 ± 0.98
	*H*	1.986	1.111	0.946	1.862
	*p*	0.575	0.774	0.814	0.601

Working years	1–5	2.70 ± 0.91	2.67 ± 0.83	2.70 ± 0.80	2.76 ± 1.03
	6–10	2.62 ± 0.89	2.61 ± 0.88	2.78 ± 0.83	2.68 ± 0.96
	11–15	2.77 ± 0.90	2.66 ± 0.78	2.65 ± 0.81	2.59 ± 0.88
	16–20	2.75 ± 0.78	2.81 ± 0.92	2.80 ± 0.90	2.92 ± 1.15
	> 20	2.69 ± 0.90	2.54 ± 0.76	2.73 ± 0.84	2.84 ± 0.93
	*F*	0.358	0.912	0.414	1.153
	*p*	0.838	0.457	0.798	0.331

The number of night shifts per month	0	2.79 ± 0.87	2.64 ± 0.80	2.67 ± 0.84	2.76 ± 0.91
	1–4	2.63 ± 0.85	2.66 ± 0.83	2.77 ± 0.83	2.81 ± 1.08
	5–8	2.68 ± 0.92	2.70 ± 0.85	2.73 ± 0.83	2.67 ± 1.01
	> 8	2.73 ± 0.88	2.58 ± 0.86	2.74 ± 0.80	2.81 ± 0.99
	*H*	1.986	1.111	0.946	1.862
	*p*	0.575	0.774	0.814	0.601

The number of patients rescued per month	0	2.68 ± 0.88	2.60 ± 0.82	2.64 ± 0.85	2.64 ± 1.00
	1–3	2.67 ± 0.87	2.67 ± 0.86	2.74 ± 0.79	2.77 ± 0.99
	4–6	2.86 ± 0.92	2.66 ± 0.79	2.89 ± 0.90	2.81 ± 1.00
	6–10	2.76 ± 1.13	2.61 ± 0.66	2.84 ± 0.94	2.91 ± 1.15
	> 10	2.71 ± 0.89	3.09 ± 0.90	2.60 ± 0.72	2.85 ± 1.12
	*H*	1.635	3.302	3.336	2.427
	*p*	0.802	0.509	0.503	0.658

Educational level	Junior college degree or below	2.43 ± 0.80	2.43 ± 0.61	2.34 ± 0.54	2.27 ± 0.86^a^
	Undergraduate education	2.72 ± 0.89	2.68 ± 0.84	2.75 ± 0.83	2.76 ± 1.00^ab^
	Postgraduate education or above	2.68 ± 0.89	2.44 ± 0.83	2.75 ± 0.81	3.03 ± 0.97^b^
	*H*	1.993	3.055	5.000	6.171
	*p*	0.369	0.217	0.082	0.046^∗^

Marital status	Unmarried	2.71 ± 0.90	2.60 ± 0.81	2.70 ± 0.79	2.76 ± 1.01
	Married	2.70 ± 0.88	2.69 ± 0.85	2.74 ± 0.85	2.74 ± 0.99
	*t*	0.076	−1.134	−0.572	0.184
	*p*	0.939	0.257	0.568	0.854

Number of children	0	2.74 ± 0.90	2.65 ± 0.85	2.72 ± 0.81	2.77 ± 1.03
	1	2.72 ± 0.88	2.57 ± 0.81	2.76 ± 0.84	2.72 ± 0.99
	≥ 2	2.62 ± 0.87	2.77 ± 0.84	2.70 ± 0.83	2.74 ± 0.96
	*H*	1.178	3.209	0.451	0.127
	*p*	0.555	0.201	0.798	0.938

*Note:* Different lowercase letters (a, b) indicate significant differences between pairwise groups. *H* values are from Kruskal–Wallis tests; *F* values are from one‐way ANOVA; *t* values are from independent‐sample *t* tests.

^∗^ *p* < 0.05.

## 4. Discussion

### 4.1. The Significance of the Chinese Version of IGRS

With the aging of the population and the intensifying global shortage of nurses [40], nurses’ workload continues to increase, accompanied by high work pressure [41], especially in China. The number of nurses is seriously insufficient compared with China’s huge population base, which makes the daily workload of Chinese clinical nurses heavier than that of clinical nurses in any other country [42]. The nursing work environment in our country is marked by high levels of stress, an excessive clinical workload, and significant occupational pressure, all of which pose a serious threat to the mental well‐being of nurses. In addition, the mental health status of nurses became a more severe issue during the COVID‐19 pandemic. Not only does the psychological state of nurses directly influence their own health, but it also affects the quality of medical care provided for their patients in a hospital setting [43]. Some studies showed that the most common psychological problems among nurses were depression, anxiety, burnout, and post‐traumatic stress disorder [44, 45]. Guilt, as a common negative emotion, is an essential factor leading to these mental health problems [46]. Guilt has a relatively high prevalence among nurses. Studies have shown that nurses often felt guilty due to work pressure, social stigmatization, and moral dilemmas [11, 47, 48]. It not only affected nurses’ mental health but might also affect their work efficiency and career satisfaction. Given that nurses’ psychological conditions were closely tied to the quality of care and patients’ recovery, it became essential to deal with guilt in nurses. However, in China, nurses’ interpersonal guilt has not received sufficient attention yet. Until now, there has been a gap in research on nurses’ guilt in China.

Therefore, it is necessary to develop a guilt assessment tool suitable for Chinese culture. The IGRS‐20, which was developed by Jessica Leonardi et al. [18], assesses the interpersonal guilt from four dimensions: survivor guilt, omnipotent responsibility guilt, self‐hate, and burdening guilt. This scale is more accurate at measuring interpersonal guilt and complies with China’s emphasis on national mental health. It can be used to evaluate the interpersonal guilt of nurses in China and enhance their awareness of guilt. It supplies a reliable assessment tool that helps to gain a further in‐depth and comprehensive understanding of the levels of guilt among nurses and how they influence nurses.

### 4.2. The Scientific Nature of the Chinese Localization Process of IGRS

This study was carried out by a professional team, which meticulously adhered to the Brislin translation model to perform translation, back‐translation, and cultural adaptation, ensuring the highest standards of quality and accuracy in the process [34]. Considering China’s medical and cultural context, the research team continuously revised and improved the Chinese version’s expressions, guaranteeing the equivalence of the scale translation in content, semantics, and concepts. The scale’s understandability and difficulty level were appraised from the nurses’ vantage point by means of a preliminary experiment. The entire process of the scale translation and the examination of its reliability and validity were stringently in accordance with the principles of being scientific, precise, professional, and normative, thereby ensuring the high quality of the Chinese localization of the scale and the excellent adaptability of the Chinese version of IGRS.

### 4.3. Reliability and Validity Evaluation of the Chinese Version of IGRS

In this study, a reliability evaluation of the Chinese version of the IGRS was carried out from three aspects, namely, internal consistency reliability, test–retest reliability, and split‐half reliability. The results showed that Cronbach’s α coefficient of the Chinese version of the IGRS was 0.833, indicating that the scale had good internal consistency and high credibility. The Cronbach’s alpha in this study was higher than that of the original version, perhaps indicating the differences in the learning environment and cultural background of Chinese clinical nurses. The test–retest reliability was also satisfactory, demonstrating the cross‐time stability of the Chinese version of the IGRS. Furthermore, the split‐half reliability fell within an acceptable range, which suggested that the scale boasted a high level of stability. Consequently, the Chinese version of IGRS had shown satisfactory reliability among Chinese clinical nurses.

The validity of a scale is composed of content validity and structural validity. For the Chinese version of the IGRS in this study, its I‐CVI ranged from 0.833 to 1.000, and its S‐CVI was 0.981, both lying within the reference value range [49]. It was indicated by them that good content validity was possessed by this scale. Accordingly, the results indicated that the items of the scale could reflect the content being measured more satisfactorily. In the EFA, four common factors were extracted in this study. After deleting Items 11 and 13, the cumulative variance contribution rate was 62.02%, which was consistent with the common factors in the original scale. This study further confirmed the structure of the Chinese version of IGRS by means of CFA. The results of the CFA in this study revealed that *X* ^2^/df was no more than 3, RMSEA was under 0.08, while other relative fitting indices exceeded 0.90. Besides, the fitting value reached the ideal fitting benchmark [50]. The results of further CFA suggested that the structure of the scale was scientific and stable and boasted good structural validity.

### 4.4. The Cultural Adaptability of the Chinese Version of IGRS

Given that diverse cultures possessed distinct understandings of interpersonal guilt, during the process of cross‐cultural adaptation, this study adhered stringently to the viewpoints of experts and the outcomes of preliminary experiments. In addition, after numerous discussions and repeated revisions among team members, the final study results indicated a deviation in the number of IGRS items between the Chinese version and the original scale. We decided to delete items 11 and 13, and the final scale included 18 items. The adjusted scale exhibited enhanced measurement accuracy when applied to Chinese clinical nurses. The modification of the entries in the Chinese version of IGRS was primarily attributed to two factors. The modification of the entries in the Chinese version of IGRS was primarily attributed to two factors. Foremost, Confucianism, serving as the nucleus and quintessence of China’s traditional culture, had exerted a profound and far‐reaching influence on Chinese society. Within this philosophical framework, filial piety held a pivotal position in Chinese Confucian thought and was esteemed as one of the most fundamental and indispensable moral tenets [51]. Filial piety accentuated the respect, obedience, and care that children ought to bestow upon their parents [52]. As a result, it was a commonly held belief among the Chinese that visiting parents and honoring their wishes constituted inherent responsibilities and obligations. Secondly, in China, the social structure had long been centered around the family unit, with extremely close‐knit bonds among family members [53]. Reuniting with parents was a prevailing social norm, and respecting one another’s volitions during interactions had been deeply ingrained as a customary practice. Retaining Items 11 and 13 might potentially give rise to inaccurate measurement outcomes. Hence, the decision to delete them had been made.

### 4.5. Practical Value and Characteristics of the Chinese Version of IGRS

The research results showed that the Chinese version of IGRS had highly satisfactory reliability and validity. The Chinese version of IGRS contains a total of 4 dimensions and 18 items. With a moderate number of items, the scale featured clear, specific, and highly targeted content that was readily comprehensible. The time required to complete the scale is 5 to 7 min, which is a moderate length of time, and it demonstrates good practicality and operability. Nurse individuals are enabled to augment their cognition and profound understanding of interpersonal guilt by means of this particular scale. From the nursing management perspective, nursing leaders can use the scale scores to more precisely perceive and evaluate nurses’ interpersonal guilt at work. By means of early accurate identification and timely intervention, effective support and guidance can be provided to nurses who are prone to guilt. Considering the unique circumstances of each nurse, a multidimensional and personalized health education plan and psychological intervention strategy system are meticulously designed. This promotes the optimization of nurses’ mental health, boosts their self‐efficacy, reinforces positive psychological experiences, and ultimately improves the quality of nursing work.

### 4.6. Sociodemographic Correlates of Interpersonal Guilt Among Chinese Nurses

In exploring the associations between sociodemographic factors and interpersonal guilt among Chinese nurses, educational level emerged as the only variable significantly associated with self‐hate guilt. Specifically, nurses with a postgraduate education or above scored significantly higher on self‐hate guilt than those with a junior college degree or below. This finding is noteworthy, as it suggests that advanced educational attainment may paradoxically increase vulnerability to self‐critical guilt experiences rather than serving as a protective factor.

Several interconnected factors may explain this association. First, nurses with postgraduate degrees are frequently expected to assume responsibilities beyond routine clinical care, including research, education, and administrative duties [54]. The concurrent demands of clinical practice and academic productivity create competing pressures that may predispose highly educated nurses to perceive themselves as falling short of expectations, thereby intensifying self‐blame and guilt‐related distress. Second, the deeply rooted cultural emphasis on diligence and self‐improvement, reinforced by the highly competitive educational system, may foster internalized pressure to meet high self‐expectations. When these individuals encounter inevitable setbacks, they may be more prone to self‐critical attributions. Third, nurses with postgraduate education remain a minority within the nursing workforce [55], which may contribute to feelings of professional isolation and heightened performance anxiety due to limited peer support and role models [56].

Taken together with the above findings, the absence of significant differences across other demographic factors further supports that nurses’ interpersonal guilt is less affected by external work intensity, work department, working seniority, and family status, but is instead more closely related to individual internal professional cognition and self‐requirement shaped by long‐term academic education. Nursing managers should consider implementing hierarchical psychological interventions based on the educational level, providing more targeted cognitive adjustment training for highly educated nurses to reduce excessive self‐attribution bias and relieve self‐hate guilt, while helping nurses across all educational backgrounds maintain positive professional cognition and stable psychological status. Future research could further explore the mechanisms underlying this association through longitudinal or qualitative approaches and develop stratified intervention models tailored to different educational levels, so as to inform evidence‐based psychological support strategies for the nursing workforce.

## 5. Limitation and Strength

Several limitations of this study are worth noting and discussing. Firstly, because of the limitations in terms of time and resources, this study was only targeted at clinical nurses in Yunnan Province. Consequently, the samples may not fully represent the entire population. In the future, it is necessary to conduct more large‐sample and multi‐center studies in different provinces and medical institutions to enrich the research results. Secondly, convenience sampling was adopted in this study, and this might lead to the determination of sample units being unrepresentative.

This study translated the IGRS scale into Chinese and verified its reliability and validity among clinical nurses in China, providing an effective and accurate tool for assessing nurses’ interpersonal guilt levels in the future.

## 6. Conclusion

In this study, the Chinese version of IGRS showed good reliability and reliability, including 18 items and a four‐factor structure. It can be used to measure the interpersonal guilt level of Chinese clinical nurses. The scale provides a theoretical basis for nurses to understand and manage guilt scientifically and helps to deeply understand nurses’ mental state and take effective intervention measures to maintain mental health.

## Funding

This work was supported by the Yunnan Health Training Project of High Level Talents, Grant No. D‐2024023.

## Ethics Statement

Before completing the questionnaire, all participants were informed about the content and significance of this study and provided their informed consent. They then completed the questionnaire anonymously. All procedures were performed in accordance with the 1964 Declaration of Helsinki. This study protocol was approved by the Ethics Committee of the First Affiliated Hospital of Kunming Medical University on July 9, 2024 (Approval No. 2024L167).

## Conflicts of Interest

The authors declare no conflicts of interest.

## Supporting Information

Additional supporting information can be found online in the Supporting Information section.

## Supporting information


**Supporting Information** The supporting information includes the STROBE checklist (Strobe‐checklist.docx), documenting adherence to reporting standards for observational studies and supporting the transparency, rigor, and reproducibility of this research. It is available via the submission system.

## Data Availability

The datasets used and/or analyzed during the current study are available from the corresponding author on reasonable request.
